# Antimicrobial oxygen-loaded nanobubbles as promising tools to promote wound healing in hypoxic human keratinocytes

**DOI:** 10.1016/j.toxrep.2022.01.005

**Published:** 2022-01-28

**Authors:** Giuliana Banche, Valeria Allizond, Narcisa Mandras, Nicole Finesso, Anna Luganini, Tullio Genova, Monica Argenziano, Chiara Magnetto, Giulia Rossana Gulino, Janira Roana, Vivian Tullio, Giuliana Giribaldi, Roberta Cavalli, Rita Spagnolo, Adriano Troia, Anna Maria Cuffini, Mauro Prato

**Affiliations:** a Department of Public Health and Pediatric Sciences, University of Torino, Via Santena 9, 10126, Turin, Italy; b Oncology Department, University of Torino, Via Santena 5 bis, 10126, Turin, Italy; c Department of Life Sciences & Systems Biology, University of Torino, Via Accademia Albertina 13, 10123, Turin, Italy; d Department of Drug Science and Technology, University of Torino, Via Pietro Giuria 9, 10125, Turin, Italy; e Istituto Nazionale di Ricerca Metrologica, Strada delle Cacce, 91, 10135, Turin, Italy; f Department of Neuroscience, University of Torino, Corso Raffaello 30, 10125, Turin, Italy

**Keywords:** Nanobubbles (NBs), Methicillin-resistant *Staphylococcus aureus* (MRSA), *Candida albicans*, Keratinocytes, Chronic wounds (CWs)

## Abstract

Chronic wounds (CWs) are typically characterized by persistent hypoxia, exacerbated inflammation, and impaired skin tissue remodeling. Additionally, CWs are often worsened by microbial infections. Oxygen-loaded nanobubbles (OLNBs), displaying a peculiar structure based on oxygen-solving perfluorocarbons such as perfluoropentane in the inner core and polysaccharydes including chitosan in the outer shell, have proven effective in delivering oxygen to hypoxic tissues. Antimicrobial properties have been largely reported for chitosan. In the present work chitosan/perfluoropentane OLNBs were challenged for biocompatibility with human skin cells and ability to promote wound healing processes, as well as for their antimicrobial properties against methicillin-resistant *Staphylococcus aureus* (MRSA) and *Candida albicans*. After cellular internalization, OLNBs were not toxic to human keratinocytes (HaCaT), whereas oxygen-free NBs (OFNBs) slightly affected their viability. Hypoxia-dependent inhibition of keratinocyte migratory ability after scratch was fully reversed by OLNBs, but not OFNBs. Both OLNBs and OFNBs exerted chitosan-induced short-term bacteriostatic activity against MRSA (up to 6 h) and long-term fungistatic activity against *C. albicans* (up to 24 h). Short-term antibacterial activity associated with NB prolonged adhesion to MRSA cell wall (up to 24 h) while long-term antifungal activity followed NB early internalization by *C. albicans* (already after 3 h of incubation). Taken altogether, these data support chitosan-shelled and perfluoropentane-cored OLNB potential as innovative, promising, non-toxic, and cost-effective antimicrobial devices promoting repair processes to be used for treatment of MRSA- and *C. albicans*-infected CWs.

## Highlights

• Chitosan-shelled/perfluoropentane-filled OLNBs are innovative oxygen nanocarriers.
• OLNBs are biocompatible with human keratinocytes after cell internalization.
• OLNBs promote normoxia-like migration of hypoxic human keratinocytes.
• Chitosan-shelled OLNBs display antimicrobial activity against MRSA and *C. albicans.*
• Oxygen-loaded nanobubbles appear promising tools to treat infected chronic wounds.

## Graphical abstract

**Figure:**
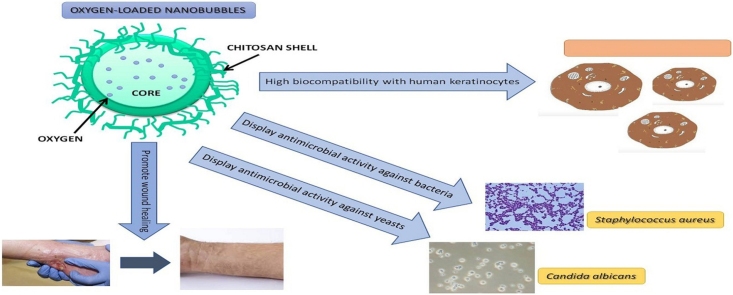

## 1 Introduction

Oxygen plays a pivotal role in achieving successful wound healing as a consequence of its increased demand for reparative processes. When oxygen is lacking for prolonged times, physiological mechanisms of wound repair are seriously compromised, allowing the injury of the skin to become chronic [1]. A chronic wound (CW) is defined as a break in skin epithelial continuity lasting more than 42 days and characterized by persistent hypoxia, exacerbated inflammation, and impaired skin tissue remodeling [[2], [3], [4]]. Their prevalence varies with age, ranging approximately from 1% in the adult population to 3–5% in >65 year-old subjects. Thus, representing a global issue costing millions of dollars per year in developed countries.

Wound healing is also complicated by microbial infections, which can cause tissue necrosis and systemic infection [5]. Microorganisms, including bacteria and yeasts, are normally sequestered on the skin surface and in case of injury they usually can trespass it only temporarily, before the wound is healed. However, in CWs the sudden gain of access to the underlying tissues by microbes becomes persistent, leading to prolonged increase of pro-inflammatory cytokines and elongation of the inflammatory phase [6]. Methicillin-resistant *Staphylococcus aureus* (MRSA) and *Candida albicans* are two frequent pathogens involved in CWs from immunocompromised or diabetic patients [[7], [8], [9]]. Antimicrobial therapies are considered the gold standard; unfortunately, in the last decades the number of resistant organisms, is mounting [10]. Limited efficacy of transdermal delivery represents an additional matter of concern. Therefore, there is an urgent need for innovative and effective therapies for the treatment of infected CWs [6,11].

In this context, different studies have investigated the potential of natural compounds coupled with nanotechnology, as an applicable knowledge, on wound healing processes [12,13]. Biopolymers such as chitosan, cellulose, or alginic acid have been shown to exert anti-inflammatory, anti-oxidant, and anti-bacterial or anti-fungal activities along with promoting collagen synthesis and deposition [12]. Recent investigations on biocompatibility and anti-microbial activity of nanoparticle-carried natural compounds have shown that they can be influenced deeply by many factors, including nanoformulation type (film, foam, or hydrogel) or ingredient concentration and nanoparticle surface-to-volume ratio [13,14]. By controlling these parameters, suitable nanoformulations can be obtained for the development of wound dressings and tissue engineering scaffolds [12].

Among the most promising candidate polysaccharides to be employed as biomaterials for the development of new nanomedical devices, chitosan has attracted attention in the field of green nanotechnology due to its natural origin, biodegradability, biocompatibility, non-toxicity, non-antigenicity, and low-cost feasibility as well as its anti-microbial, anti-tumor, anti-oxidant, anti-diabetic, and immunoenhancing properties [15,16]. Chitosan is generated after N-deacetylation of chitin, and a wide spectrum of linear chitosan molecules exist, varying for molecular weight, viscosity, and degree of deacetylation [[17], [18], [19], [20]].

In this context, we have previously carried out intensive research focusing on chitosan species or derivatives as candidate molecules to be employed for the manufacturing of the outer shell of oxygen-loaded nanobubbles (OLNBs) [[21], [22], [23], [24], [25], [26], [27]] and oxygen-loaded nanodroplets (OLNDs) [[28], [29], [30], [31], [32]]. These two innovative platforms of oxygen nanocarriers consist in the encapsulation in the inner core of oxygen-solving perfluoropentane and 2H,3H-decafluoropentane molecules, respectively.

Both OLNB and OLND delivery systems exploit the Laplace’s law for spherical surfaces, therefore, the smaller bubble’s radius, the higher differential pressure of the gas, and the faster diffusion of the gas [33]. Oxygen release from OLNBs or OLNDs either occurs spontaneously, through passive diffusion, or it can be induced upon ultrasound administration, destabilizing its shell and allowing instant release of the gas from the inner core [34]. Additionally, OLNB and OLND peculiar architecture allows for further functionalization of the carrier with additional molecules (drugs, dyes, antibodies, etc.) [30,32,35,36].

Due to such advantageous characteristics, coupled with the exceptional biochemical and pharmacological properties of chitosan, these nanocarriers appear potentially suitable for treatment of CWs. In this context, a series of *in vitro* studies has already provided consistent evidence on the effectiveness of OLNDs in counteracting the effects of hypoxia in several skin cell models [31,37,38] as well as the efficacy of free or drug-coupled NDs in promoting anti-mcrobial activity against some bacteria (*S. aureus*, *Enterococcus faecalis*, *E. faecium*) [29,32,35], yeasts (*C. albicans*) [29], and viruses (Herpes simplex virus-2) [36].

Therefore, it is interesting to study the suitability of the OLNB for CW treatment. For this reason, in the present work the OLNBs with chitosan and perfluoropentane shells were evaluated for their biocompatibility with human keratinocytes and the ability to promote wound healing processes. Furthermore, the anti-bacterial and anti-fungal properties of OLNB against clinical strains of MRSA and *C. albicans* were evaluated and the physical interactions between NBs and membranes or cell walls were investigated.

## 2 Materials and methods

### 2.1 Materials, instruments, and software

Materials: plastics were from Costar (Corning, USA), Jet Biofil (Guangzhou, China) and VWR (Radnor, Pennsylvania); 0.9 % sodium chloride saline solution was from Baxter (Bloomington, USA); 96 % ethanol was from Carlo Erba (Cornaredo, Italy); Epikuron 200® (95 % soya phosphatidylcholine) was from Degussa (Hamburg, Germany); palmitic acid, perfluoropentane, and polyvinylpyrrolidone (PVP) were from Fluka (Buchs, Switzerland); Mannitol Salt Agar (MSA), Trypticase Soy Broth or Agar (TSB, TSA), and Sabouraud dextrose (SAB) broth and agar were from Oxoid SpA (Rodano, Italy); cell culture RPMI 1640 medium and propidium iodide (PI) were from Invitrogen-Thermo Fisher Scientific Inc. (Carlsbad, USA); cryovials were from Microbank, BioMérieux (Marcy-l’Etoile, France); CellTiter-Glo® was from Promega (Madison, USA); cell culture inserts were from Ibidi (Planegg, Germany); any other materials not detailed above were from Sigma-Aldrich (Saint Louis, USA).

Instruments: ultrapure water was obtained using a 1–800 Millipore system (Molsheim, France); Ultra-Turrax SG215 homogenizer was from IKA (Staufen, Germany); Delsa Nano C analyzer was from Beckman Coulter (Brea, USA); HQ40d model oxymeter was from Hach Lange (Derio, Spain); AE31 optical microscope was from Motic (Xiangan Qu, China); XDS-3FL optical microscope was from Optika (Ponteramica, Italy); CM10 transmission electron microscopy (TEM) was from Philips (Eindhoven, The Netherlands); Synergy HT microplate reader was from Bio-Tek Instruments (Winooski, USA); Olympus Fluoview 200 laser scanning confocal system and inverted IX70 Olympus microscope was from Olympus America Inc. (Center Valley, USA); plan flour fluorescent microscope was from Nikon (Minato, Japan).

Software for statistical analysis and scientific graphing: software SPSS 16.0 for Windows was from SPSS Inc. (Chicago, USA) and software Graphpad Prism version 6.00 for Windows was from Graphpad Software (San Diego, USA).

### 2.2 Manufacturing of oxygen-loaded nanobubble and control formulations

OLNB formulations were prepared as described previously [34]. Briefly, a pre-emulsion was obtained adding 300 mL of an ethanol solution containing Epikuron® 200 and 1% w/v palmitic acid to perfluoropentane under magnetic stirring. After addition of 4.8 mL of phosphate buffered saline (PBS), the system was homogenized for 2 min using an Ultra-Turrax SG215 homogenizer. Thereafter, oxygen was added to the suspension for 2 min. Then, 0.14 % w/v aqueous solution of medium weight (MW) chitosan (pH 4.5) was added drop-wise under magnetic stirring. To produce oxygen-free nanobubbles (OFNBs) the addition of oxygen was skipped. Similarly, oxygen-saturated solution (OSS) was prepared without adding chitosan and perfluoropentane. For selected experiments (confocal microscopy studies), NBs were conjugated with fluorescein isothiocyanate (FITC) solution overnight (FITC concentration: 10 % for studies on human cells and 7% for studies on microbial cells).

### 2.3 Nanobubble in vitro characterization

The morphology of NB formulations was assessed by optical microscopy and by TEM, as described previously [34,21,39]. NB suspensions were dropped onto a Formwar-coated copper grid and air-dried before observation.

The average diameters, polydispersity indexes and zeta potentials of NBs were determined by dynamic light scattering, as described previously [34,21,39]. A monochromatic light source (laser ray) was shot through a polarizer into each sample, previously diluted in deionized water before measurement. The scattered light then went through a second polarizer where it was collected by a photomultiplier and the resulting image (speckle pattern) was projected onto a screen. This process was repeated at short time intervals and the resulting set of speckle patterns were analyzed by an autocorrelator comparing the intensity of light at each spot over time. The most important use of the autocorrelation function (also known as photon correlation spectroscopy) is its use for size determination. The polydispersity index indicates the size distribution within a NB population. For the zeta potential determination, samples from each formulation were placed in an electrophoretic cell, where an electric field of 15 V/cm was applied. Each sample was analyzed at least in triplicate. The measured electrophoretic mobility was converted into zeta potential value using the Smoluchowsky equation [40].

### 2.4 Nanobubble sterilization

NB formulations were sterilized through UV-C exposure for 20 min. Thereafter, UV-C-treated materials were incubated with cell culture RPMI 1640 medium in a humidified CO_2_/air-incubator at 37 ° C up to 72 h, not displaying any signs of microbial contamination when checked by optical microscopy.

### 2.5 Human cells, bacteria, and yeasts

HaCaT cell line, immortalized from a 62-year old Caucasian male donor [41], was used as a source of human keratinocytes. HaCaT cells were grown in a humidified CO_2_/air-incubator at 37 ° C as monolayers in Dulbecco’s Modified Eagle Medium - high glucose (DMEM HG) supplemented with 10 % foetal bovine serum (FBS), 100 U/mL penicillin, 100 μg/mL streptomycin and 2 mM l-glutamine.

Clinical strains of MRSA and *C. albicans* previously isolated from human ulcerated wounds of patients from Infermi Hospital (Biella, Italy) were cultured at 37 ° C on MSA and SAB agar, respectively. Species identification were performed using the MALDI-TOF technology (MALDI Biotyper Systems Bruker; Billerica, MA). The organisms represented a selected sample, due to their high frequency as pathogens involved in CWs.

Young colonies (18−24 h) were picked up to approximately 3−4 McFarland standard and inoculated into cryovials containing both cryopreservative fluid and porous beads to allow microorganisms to adhere. After inoculation, cryovials were kept at -80 ° C for extended storage.

### 2.6 3-(4,5-dimethylthiazol-2-yl)-2,5-diphenyltetrazolium bromide assay

Cell viability was evaluated using 3-(4,5-dimethylthiazol-2-yl)-2,5-diphenyltetrazolium bromide (MTT) assay. Cells were seeded in 96-multi-well plates (10^4^ cells/well) in their supplemented cell culture medium and incubated in a humidified CO_2_/air-incubator at 37 ° C overnight to allow cellular adhesion. Then, cells were incubated in their supplemented medium for 24 h without or with 10 % v/v OLNBs or OFNBs, either in normoxic (20 % O_2_) or hypoxic (1% O_2_) conditions, in a humidified CO_2_/air-incubator at 37 ° C. After 24 h, cells reached about 90 % of confluence. Medium was discarded and 20 μl of 5 mg/mL MTT in PBS were added to cells for 3 additional hours at 37 ° C. After plate centrifugation and cell supernatant discarding, the dark blue formazan crystals were dissolved using 100 μl of sodium dodecyl sulfate (SDS). The plates were read on Synergy HT microplate reader at a test wavelength of 550 nm and at a reference wavelength of 650 nm. Data are expressed as percentages of viability.

### 2.7 Lactate dehydrogenase assay

The potential cytotoxic effects of NBs were measured as the release of lactate dehydrogenase (LDH) from cells into the extracellular medium. Cells were seeded in 6-multi-well plates (3 × 10^5^ cells/well) in 2 mL/well of supplemented cell culture medium and incubated in a humidified CO_2_/air-incubator at 37 ° C overnight to allow cellular adhesion. The day after, cells were incubated in absence or presence of 10 % v/v OLNBs or OFNBs either in normoxic (20 % O_2_) or hypoxic (1 % O_2_) conditions, in a humidified CO_2_/air-incubator at 37 ° C. After 24 h, cells reached about 90 % of confluence and 1 mL of cell supernatants were collected and centrifuged at 13,000 *g* for 30 min. Cells were washed with fresh medium, detached with scraper, washed with PBS, resuspended in 1 mL of 82.3 mM triethanolamine solution, pH 7.6 (TRAP), and sonicated on ice with a 10 s burst. Five μl of cell lysates and 50 μl of cell supernatants were diluted with TRAP and supplemented with 0.5 mM sodium pyruvate and 0.25 mM nicotinamide adenine dinucleotide reduced form (NADH) (300 μl as a final volume). The reaction was monitored by measuring the absorbance at 340 nm (37 ° C) with Synergy HT microplate reader. After determining the intracellular and extracellular LDH activities, expressed as μmol of oxidized NADH/min/well, cytotoxicity was eventually calculated as the net ratio between extracellular and total (intracellular + extracellular) LDH activities.

### 2.8 Adenosine triphosphate assay

The CellTiter-Glo® Luminescent Cell Viability Assay is a homogeneous method for determining the number of viable cells in culture based on quantification of detected adenosine triphosphate (ATP), an indicator of metabolically active cells. Briefly, were seeded in 96 multi-wells cells (10^3^/well) in their supplemented cell culture medium and incubated in a humidified CO_2_/air-incubator at 37 ° C overnight to allow the cellular adhesion. The day after, cells were incubated in absence or presence of 10 % v/v OLNBs or OFNBs either in normoxic (20 % O_2_) or hypoxic (1% O_2_), in a humidified CO_2_/air-incubator at 37 ° C. After 24 h, cells reached about 90 % of confluence and CellTiter-Glo® Luminescent Cell Viability Assay was performed following manufacturer’s instructions. ATP production was expressed as relative light units detected through the assay.

### 2.9 Confocal microscopy analyses

HaCaT cells (6 × 10^4^ cells/well) were seeded and incubated under normoxic condition (20 % O_2_) in humidified CO_2_/air-incubator at 37 ° C overnight. Then, cells were left untreated or treated with 10 % v/v FITC-labeled OLNB or OFNB suspensions for 24 h in normoxia. After incubation, human cells were fixed with 1% paraformaldehyde (PFA) PBS solution for 15 min. Then cells were incubated with 15 μg/mL PI to visualize nucleic acids. Confocal images were acquired by a LSM710 inverted confocal laser scanning microscope equipped with a Plan-Neofluar 63 × 1.4 oil objective that allowed a field view of at least 5 cells. A wavelength of 488 nm was used to detect OLNBs and of 460 nm to detect the labeled nuclei. The acquisition time was 400 ms. Confocal images were taken using FITC and tetramethylrhodamine (TRITC) filters.

MRSA cells [10^9^ colony forming units (CFUs)/mL] were incubated without or with 10 % v/v FITC-labeled OLNBs or OFNBs for 3 and 24 h with agitation at 37 ° C. At each incubation time, bacteria and different formulation suspensions (50 μl) were transferred on glass slides, heat-fixed, and subsequently stained with 5 μg/mL PI in humid chamber at 37 ° C for 15 min. Bacteria were fixed with mounting solution and covered by cover slips. Confocal fluorescent images were taken using FITC and TRITC filters.

*C. albicans* cells (10^8^ CFUs/mL) were stained with 10 μg/mL PI and incubated without or with 10 % v/v FITC-labeled OLNBs or OFNBs, for 3 and 24 h with agitation at 37 ° C. At each incubation time, 100 μl suspensions from yeasts and different formulations were transferred on glass slides. Yeasts were fixed with mounting solution and covered by cover slips. Confocal fluorescent images were taken using FITC and TRITC filters.

### 2.10 Wound healing assay

Cell abilities to perform wound healing were assessed through a specific *in vitro* biological assay, commonly known as scratch assay. Briefly, cells were seeded into cell culture Ibidi inserts (2 × 10^4^ cells/insert), a specific type of supports composed by a 2-well silicone insert with a defined cell-free gap. Cells were incubated for 24 h, to allow cellular adhesion. Then, supports were removed and cells were incubated in absence or presence of 10 % v/v OLNB or OFNB suspensions or OSS in normoxic (20 % O_2_) or hypoxic (1% O_2_) conditions for 16 h. Live imaging was then performed using Nikon Plan Fluor fluorescent microscope (magnification 20X). Pictures were taken using Nikon Eclipse TI-E objective. ImageJ (1.48 v) was used to analyze pictures. Briefly, wound areas were delimited using the free-hand selection tool; then, the measurement tool was employed to edit the data window listing the area in μm^2^ for each scratch.

### 2.11 Microbiological assays

MRSA (10^4^ CFUs/mL) were incubated in TSB alone (growth control) or with 10 % v/v OSS, 0.139 % MW chitosan solution, and OFNB or OLNB formulations in sterile sampling tubes for 2, 3, 4, 6, and 24 h at 37 ° C. At each incubation time, serial ten-fold dilutions from each sample were prepared in 0.9 % NaCl saline solution and 100 μl of each dilution was spread on TSA to determine the number of CFU/mL and incubated in a humidified CO_2_/air-incubator at 37 ° C for 24 h.

*C. albicans* (10^5^ CFUs/mL) were incubated in SAB broth and experiments were performed as described above for bacteria. At each incubation time, each dilution was spread on SAB agar to determine the number of CFU/mL and incubated at 37 ° C for 24 h.

### 2.12 Statistical analysis

Each condition was performed at least in duplicate for every experiment. At least three independent experiments were performed for every investigational study. Numerical data are shown as means ± standard errors of the means (SEM) for inferential results or as means ± standard deviations (SD) for descriptive results [42]. Imaging data are shown as representative pictures. All data were analyzed for significance by a one-way analysis of variance (ANOVA) followed by Tukey’s post hoc test.

## 3 Results

### 3.1 Nanobubble in vitro characterization

After manufacturing, OLNBs and OFNBs were characterized for morphology by optical microscopy and TEM, as well as for average diameters, polydispersity indexes, and zeta potentials by dynamic light scattering.

**Fig. 1:**
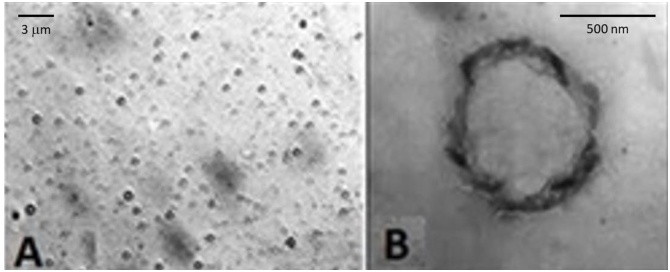
**NB morphology and structure.** OLNBs were checked for morphology by optical microscopy and TEM. Results are shown as representative images from ten different preparations. Panel A. OLNB image by optical microscopy. Magnification: 630 × . Panel B. OLNB image by TEM. Magnification: 52000 × .

As emerged from checking by optical microscopy (Fig. 1A) and TEM (Fig. 1B), NBs displayed spherical shapes, with TEM analysis also highlighting the typical core shell-structure. Further light scattering analyses revealed NB sizes to be in the nanometer range, with average diameters of ∼700 nm for OLNBs and ∼300 nm for OFNBs. Moreover, NBs displayed cationic zeta potentials with values around +39 mV. Polydispersity indexes for OLNB and OFNB suspensions were 0.19 and 0.10, respectively. The physico-chemical characteristics of all NB formulations tested in this study are summarized in Table 1.

**Table 1:** **Physico-chemical characterization of OLNBs and OFNBs**. OLNB/OFNB formulations were characterized for diameters, polydispersity indexes, and zeta potentials by dynamic light scattering. Results are shown as means ± SD from ten different preparations for each formulation.

Nanocarrier	OLNB	OFNB
Outher shell plysaccharide	MW chitosan	MW chitosan
Inner core fluorocarbon	perfluoropentane	perfluoropentane
Fluorocarbon boiling point	29 °C	29 °C
Diameter (nm ± SD)	745.20 ± 117.89	320.40 ± 100.90
Polydispersity index	0.19	0.10
Zeta potential (mV ± SD)	+39.20 ± 1.00	+38.65 ± 1.00

### 3.2 Biocompatibility of nanobubbles with normoxic and hypoxic human keratinocytes

**Fig. 2:**
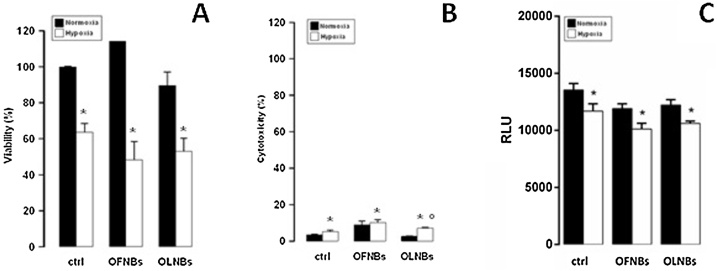
**Effects of hypoxia and NBs on human keratinocyte viability, health, and metabolism.** HaCaT cells (1.6 × 10^5^ cells/mL for MTT studies, 5 × 10^5^ cells/mL for LDH studies, and 5 × 10^5^ cells/mL for ATP studies) were left untreated or treated with OLNBs and OFNBs for 24 h in normoxia (20 % O_2_, black columns) or hypoxia (1% O_2_, white columns). After collection of cell supernatants and lysates, cell viability percentage was measured through MTT assay (panel A), cytotoxicity percentage through LDH assay (panel B), and ATP production through ATP assay (panel C). Results are shown as means + SEM from three independent experiments. Data were also evaluated for significance by ANOVA: * vs normoxic control cells: *p* < 0.05; ° vs hypoxic control cells: *p* < 0.05.

The biocompatibility of OLNBs and OFNBs with normoxic and hypoxic human keratinocytes was evaluated by using a series of complementary biochemical assays. In particular, cell viability was checked by MTT assay, treatment cytotoxicity was analyzed by LDH assay, and cell metabolic activity was measured by ATP assay. As shown in Fig. 2 (Panels A, B, and C: results from MTT, LDH, and ATP assays, respectively), hypoxia displayed significant toxicity, affecting HaCaT cell viability and metabolism. However, the values obtained in the presence of OLNB treatment either in normoxia or in hypoxia were not significantly different in comparison with their respective controls. A slight toxicity of OFNDs was observed after measuring LDH release, but cell viability and metabolic activity were not affected significantly, as checked through MTT and ATP assays.

### 3.3 Mechanical interaction between nanobubbles and human keratinocytes

**Fig. 3:**
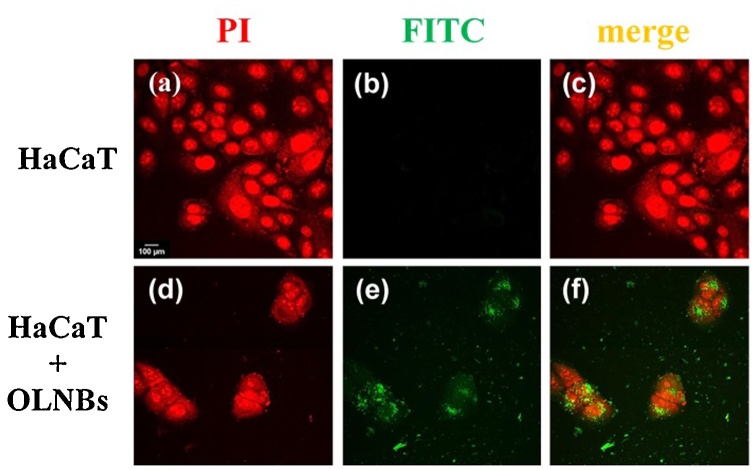
**OLNB internalization by human keratinocytes.** HaCaT cells (6 × 10^4^ cells/mL) were left untreated or treated with FITC-labeled OLNBs for 24 h in normoxia (20 % O_2_). After PI staining, cells were checked by confocal microscopy. Results are shown as representative images from three independent experiments. Panels a-c (red, green, merged): control cells; panels d-f (red, green, merged): OLNB-treated cells. Red: cell nuclei after PI staining. Green: FITC-labeled OLNBs. Magnification: 60×.

The mechanical interaction between OLNBs and human keratinocytes was investigated through analysis by confocal microscopy. As shown in Fig. 3 (panel f), OLNBs appeared to be avidly internalized by human keratinocytes after 24 h of incubation in normoxic conditions. Similar results were obtained also for OFNBs (data not shown).

### 3.4 Effects of hypoxia and oxygen-loaded nanobubbles on the migration of human keratinocytes

**Fig. 4:**
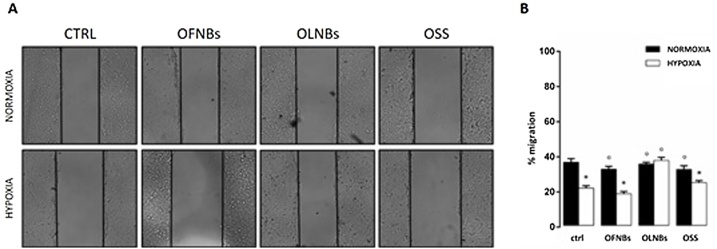
**Effects of hypoxia and OLNBs on the migration and wound healing abilities of human keratinocytes.** HaCaT cells (3 × 10^5^ cells/mL) were seeded in two confluent monolayers, divided by a scratch of 500 μm, and incubated for 16 h in normoxia (20 % O_2_) or hypoxia (1% O_2_) with/without 10 % v/v OLNBs as well as OFNBs or OSS. Thereafter, scratch lengths were photographed and measured. Panel A: representative images. Panel B: means + SEM of scratch lengths. Results are from three independent experiments performed in triplicates. Data were also evaluated for significance by ANOVA: * vs normoxic untreated cells: *p* < 0.01; ° vs hypoxic untreated cells: *p* < 0.01.

HaCaT cell abilities to migrate and to promote wound healing under hypoxic conditions after treatment with OLNBs, as well as OFNBs or OSS, were evaluated through scratch assay. As shown in Fig. 4, hypoxia slowed down the migration of human keratinocytes with respect to normoxic conditions. Additional treatment with OLNBs fully abrogated hypoxia-dependent dysregulation of cell migration restoring a normoxia-like cell migratory behavior, whereas OFNBs and OSS did not.

### 3.5 Interaction between nanobubbles and methicillin-resistant *Staphylococcus aureus*

**Fig. 5:**
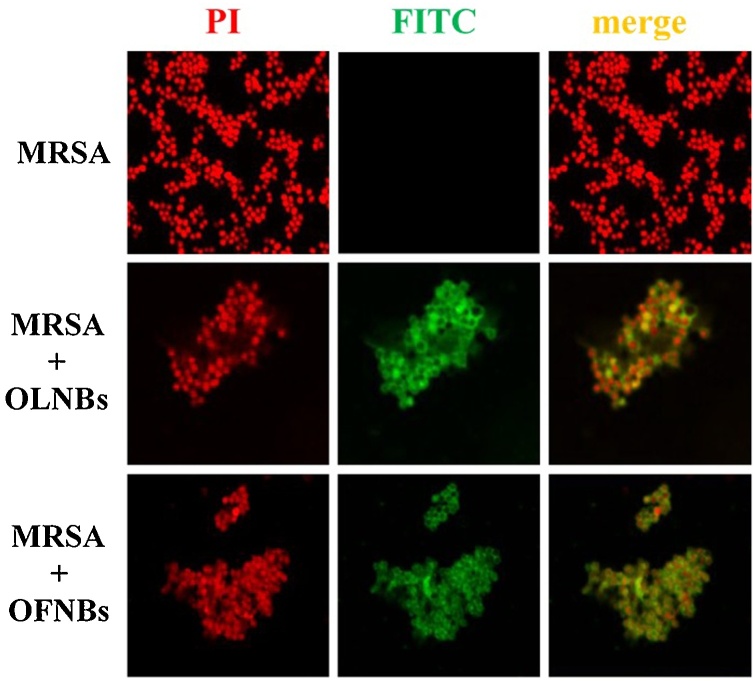
**NB adhesion to MRSA bacterial wall.** MRSA (10^9^ CFUs/mL) were left alone or incubated with 10 % v/v FITC-labeled OLNBs/OFNBs for 24 h. After staining bacteria with PI, confocal fluorescent images were taken using FITC and TRITC filters. Data are shown as representative images from three independent experiments. Red: PI. Green: FITC. Magnification: 100 × .

The interaction between OLNBs or OFNBs and MRSA was investigated through analysis by confocal microscopy. After 3 h of incubation, NBs seemed to physically interact with MRSA wall (data not shown). After 24 h of incubation, both OLNBs and OFNBs appeared to still adhere to the bacterial wall, without being internalized by MRSA (Fig. 5).

### 3.6 Antibacterial activity of free chitosan and chitosan-shelled nanobubbles on methicillin-resistant *Staphylococcus aureus*

**Fig. 6:**
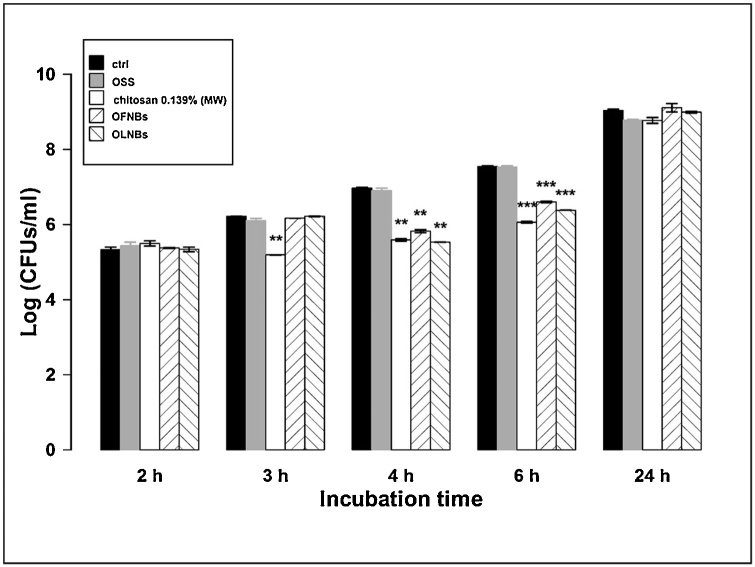
**Antibacterial activity of chitosan and chitosan-shelled NBs on MRSA.** MRSA (10^4^ CFUs/mL) were incubated alone or with 10 % v/v OSS, free MW chitosan solution (0.139 % m/v), OFNB suspension, or OLNB suspension in sterile conditions at 37 °C and their growth was monitored for 2, 3, 4, 6 and 24 h. At each incubation time, the samples were spread on TSA agar medium to determine the CFUs/mL. Results are shown as means ± SEM from three independent experiments and expressed as Log CFUs/mL. Data were evaluated for significance by ANOVA: vs controls: 3-4 h, ** *p* < 0.01; 6 h, *** *p* = 0.0001.

The antibacterial properties of NBs against MRSA were evaluated through a microbiological assay. As shown in Fig. 6, both OLNBs and OFNBs, as well as free chitosan solution, significantly inhibited MRSA growth up to 6 h of incubation, especially between 4 and 6 h, whereas OSS did not affect MRSA growth. At these observational time-points, no significant differences were observed among treatments with OLNBs, OFNBs, and chitosan alone. The bacterial growth did not appear to be affected by any treatments after 24 h of incubation. OSS did not affect bacterial growth at any times.

### 3.7 Mechanical interaction between nanobubbles and *Candida albicans*

**Fig. 7:**
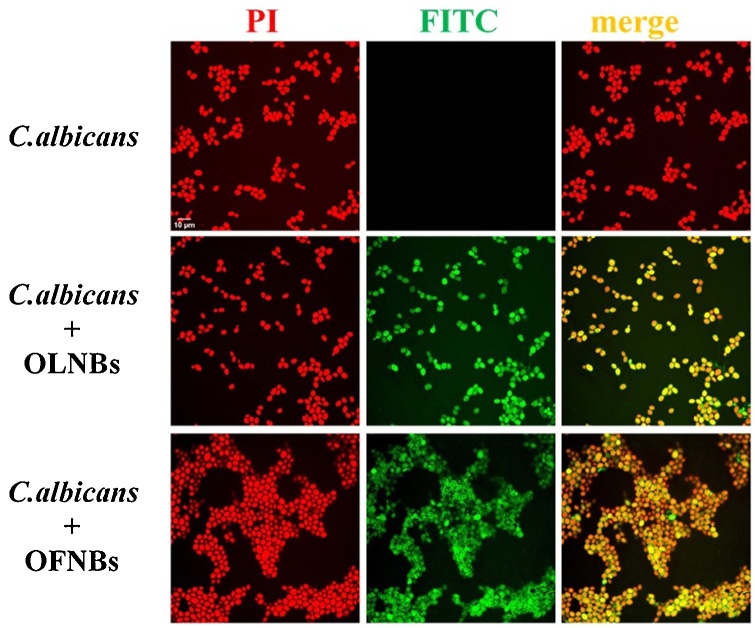
**NB internalization by *C. albicans*.***C. albicans* (10^8^ CFUs/mL) were left alone or incubated with 10 % v/v FITC-labeled OLNBs or OFNBs for 3 h. After staining yeasts with PI, confocal fluorescent images were taken using FITC and TRITC filters. Data are shown as representative images from three independent experiments. Red: PI. Green: FITC. Magnification: 100 × .

The mechanical interaction between OLNBs or OFNBs and *C. albicans* was investigated through analysis by confocal microscopy. As shown in Fig. 7, both formulations appeared to be avidly uptaken and internalized by yeasts already at the earlier observational time-point (3 h). Consistently, OLNB or OFNB internalization by yeasts was also observed at the later time-point (24 h) of incubation (data not shown).

### 3.8 Antifungal activity of free chitosan and chitosan-shelled nanobubbles on *Candida albicans*

**Fig. 8:**
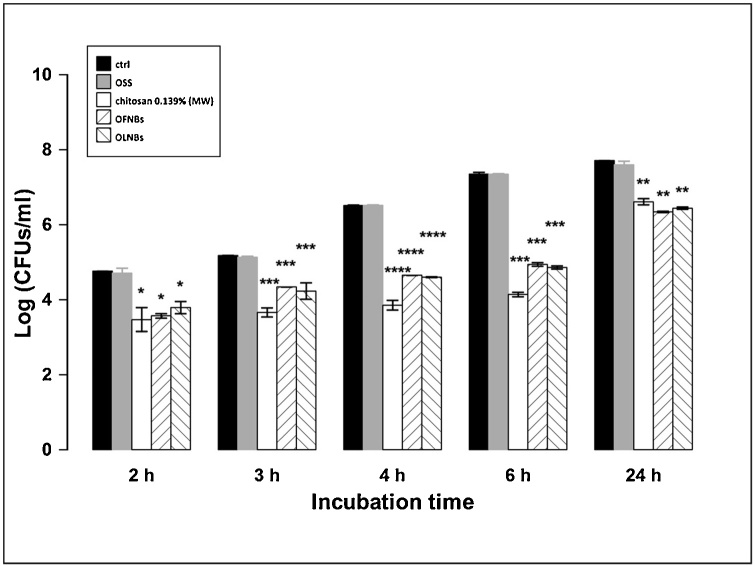
**Antifungal activity of free chitosan and chitosan-shelled NBs on *C. albicans*.***C. albicans* (10^5^ CFUs/mL) were incubated alone or with 10 % v/v OSS, free MW chitosan (0.139 % m/v) solution, OFNB suspension, or OLNB suspension in sterile conditions at 37 °C and their growth was monitored for 2, 3, 4, 6 and 24 h. At each incubation time, the samples were spread on SAB agar medium to determine the CFUs/mL. Results are shown as means ± SEM from three independent experiments and expressed as Log CFUs/mL. Data were evaluated for significance by ANOVA: vs controls: 2 h, * *p* < 0.05; 3-6 h, *** *p* = 0.0001; 4 h, **** *p* < 0.0001; 24 h, ** *p* < 0.01.

The antifungal properties of NBs against *C. albicans* were evaluated through a microbiological assay. As shown in Fig. 8, both chitosan-shelled OLNBs and OFNBs, as well as free chitosan solution, significantly inhibited *C. albicans* growth up to 24 h of incubation, with no significant differences being observed among these three treatments at any observational time-points. On the contrary, OSS did not affect fungal growth at any times.

## 4 Discussion

The present work aimed at challenging chitosan-shelled NBs for their biocompatibility with human keratinocytes, as well as for their ability to counteract the effects of hypoxia on keratinocyte migratory phenotype during wound healing, and for their anti-microbial properties against MRSA and *C. albicans*, typically infecting CWs.

NBs, have been prepared as aqueous nanosupensions and were manufactured either with or without oxygen in the inner core (OLNBs and OFNBs, respectively). MW chitosan was employed as component of the polysaccharidic shell while perfluoropentane was chosen as oxygen-solving perfluorocarbon for the inner core. The choice of the fluorocarbon to be inserted within the core is crucial, as it must be able to bind and to release oxygen easily and effectively, greatly improving its delivery into hypoxic environments. The chemical skeleton of perfluoropentane satisfies this prerequisite, since it presents twelve fluorine atoms which allow to establish van der Waals bonds with biatomic oxygen molecules. Consistently, previous studies from our group have confirmed perfluoropentane-containing OLNBs to display good gas capacity (∼0.45 O_2_ g/mL) along with efficient ability to release clinically relevant amounts of oxygen gradually and for sustained times to hypoxic tissues, opposite to OSS which releases almost immediately the oxygen content within the saturated solution as a whole. Results obtained from physico-chemical characterization in the present study were in line with data from literature [34,21,39], with NBs displaying average diameters in the nanometer range (∼700 nm for OLNBs and ∼300 nm for OFNBs) and zeta potentials with values around +39 mV, due to the cationic surface of chitosan shells. Zeta potential is a crucial parameter to predict the stability of nanocarriers. Cationic zeta potentials higher than +30 mV are usually required for stability of colloid systems [40]. Cationic nanocarriers have been suggested to be exquisitely suitable for topical treatments, since their positive charges interact strongly with the anionic surface of the skin [43].

The biocompatibility of OLNB and OFNB with human keratinocytes, both in normoxic and hypoxic conditions, were extremely relevant elements to be taken into consideration in our context, since the effects of hypoxia on the phenotype of human keratinocytes can be different depending on the age of the donor and skin diseases associated with hypoxia [44]. For this reason HaCaT cell line, immortalized from a 62-year old Caucasian male donor [41], was chosen as a source of human keratinocytes in our experiments. As expected, hypoxia significantly reduced viability, health, and metabolism of keratinocytes while OLNDs did not. On the contrary, and quite surprisingly, OFNBs appeared slightly cytotoxic, although they did not affect significantly cell viability and ATP production. Such cytotoxicity might be a consequence of the type of chitosan chosen for NB manufacturing (MW chitosan), as it has been reported that chitosan molecules exhibit a molecular weight-dependent negative effect on HaCaT cell viability and proliferation *in vitro* [45]. This may also be in line with results from a recent study performed on NDs, comparing the biocompatibility of several chitosan species and derivatives with human keratinocytes and suggesting low weight chitosan to be the most suitable species to be used for ND manufacturing instead of MW chitosan [28]. Therefore, future comparative studies on OFNBs shelled with different chitosan species characterized by different molecular weight may be useful to optimize their biocompatibility for treatments where oxygen is not needed. Anyway, OLNBs – the main nanocarrier undergoing investigation in the present research – did not display any toxicity and did not affect keratinocyte viability or metabolism compared to untreated controls, suggesting a protective role for oxygen on human cells. This latter evidence is extremely encouraging, as it confirms OLNBs to be suitable for the purposes of the present work, aiming at restoring the physiological phenotype of keratinocytes in hypoxic conditions as frequently found in CWs.

Further analyses by confocal microscopy were performed to discern the mechanisms of interaction between OLNBs and human cell surfaces (i.e. lack of contact, adhesion, or internalization). According to our results, OLNBs were avidly uptaken by HaCaT cells after 24 h of incubation. These data are in line with those obtained with OLNBs in monkey fibroblastoid kidney cells (Vero) [34], as well as those with OLNDs in human keratinocytes [31], endothelial cells [37], and monocytes [38]. This approach was also widely chosen to demonstrate the accumulation of chitosan in renal tubules [46] or chitosan/cyclodextrin nanoparticles internalization by Calu-3 epithelial cells [47].

In general, after interacting with human cells, nanoparticles might be engulfed in invaginations of the cell membrane and then be internalized by cells through time-, concentration-, and energy-dependent pinocytic processes [48,49]. Chitosan intracellular degradation has been hypothesized to be associated with lysosomes [50] and the mechanism of chitosan cellular binding might be a nonspecific electrostatic interaction with the negatively charged cell membrane. Therefore, evidence on OLNB cellular internalization after adhering on cell plasma membrane is not surprising.

The study of the effects of hypoxia as well as those of OLNBs on the migration of human keratinocytes by scratch assay, showed that hypoxia strongly impaired wound healing abilities of HaCaT cells. However, OLNBs restored a normoxia-like migratory phenotype. Interestingly, these effects were achieved neither by OFNBs nor by OSS, suggesting that not only oxygen presence but also its gradual release were essential.

Cell migration is a critical process during wound healing [51]. Two processes are essential during wound repair for the lesion to heal successfully. Keratinocytes should be able to detach from the underlying basal lamina, move and migrate through the new extracellular matrix within the wound [52]. This process is facilitated by matrix metalloproteinases (MMPs) and tissue inhibitors of metalloproteinases (TIMPs) [53]. All these processes are hampered by hypoxia, and dysregulated MMP/TIMP ratio is a typical feature of CWs [54]. Interestingly, chitosan-shelled OLNDs have been shown to abrogate hypoxia-dependent dysregulation of MMP/TIMP secretion, an effect specifically due to oxygen-delivering abilities of OLNDs [31]. Additionally, dextran-shelled OLNDs were reported to restore normoxia-like balances between MMP-2 and TIMP-2 as well as between MMP-9 and TIMP-1 also in hypoxic human microvascular endothelial cells [37], human monocytes [38], and human placenta explants [55]. Therefore, OLNB-dependent abrogation of the dysregulating effects of hypoxia on keratinocyte migratory phenotype as observed here may involve regulation of MMP/TIMP balances in a similar way.

NBs were also able to interact with microbial cells. OLNBs and OFNBs were shown here to interact physically with MRSA and *C. albicans*. Indeed, both NBs just adhered to bacterial cell walls, whereas they were avidly uptaken by yeasts. Although the exact molecular mechanisms underlying either event have not yet been fully elucidated, positively charged chitosan residues (protonated amine groups) have been suggested to bind to the negatively charged bacterial surface (lipoteichoic acid in Gram-positive bacteria), leading to altered membrane permeability [56]. On the other hand, *Candida* cell wall is thinner compared to that of bacteria and differs in composition for the presence of mainly mannoproteins and β-glucans [57]. Since Gram-positive bacteria differ greatly from yeasts in surface structure, this difference could justify different interactions between nanocarriers and microorganisms.

The mechanisms of interaction between NBs and microorganisms can also result in leakage of intracellular constituents causing functional impairment and even death of bacteria and yeasts. Therefore, adhesion and internalization were hypothesized to associate with short-term and long-term antimicrobial effects, respectively. This hypothesis was generally confirmed by the results obtained from microbiological assays. Indeed, whenever OLNBs or OFNBs adhered to MRSA cell wall, their anti-bacterial effects lasted up to 6 h only. On the contrary, OLNBs and OFNBs displayed long-lasting (up to 24 h) antimicrobial effects against *C. albicans* after cell internalization.

Among the compounds employed for NB manufacturing, chitosan is the only molecule exerting cytostatic activity on microorganisms. Therefore, the dependence of NB anti-microbial properties on the presence of chitosan in their shells appears likely, as supported by data obtained here with free chitosan solution alone, which mimicked the effects of NBs. In addition, it should be noted that the presence or absence of oxygen molecules within the core on nanocarriers did not affect the effectiveness of NBs, suggesting that oxygen was not involved in NB anti-microbial effects. This was confirmed by results obtained after treatment with OSS, which did not affect bacterial or fungal growth.

Available evidence from literature suggests that chitosan is able to evade the barrier properties of the outer membrane of Gram-negative bacteria under specific conditions [58]. Although the strongest anti-microbial activity of chitosan is generally observed at acidic pH, anti-microbial effects of some nanocarriers at neutral pH have been reported [59,63] Chitosan bacteriostatic effects have also been shown to vary depending on the analyzed bacterial strain, as MW of the polymer seems to enable surface interactions only [60].

As far as it concerns chitosan fungistatic activity, the uptake of chitosan-shelled NBs by yeasts may also cause cell membrane permeabilization due to chitosan binding to the surface of *Candida*. In turn, chitosan-driven membrane permeabilization might cause the inhibition of the main metabolic pathways, thus depriving cells of their energy sources [61]. Specific binding of nanocarriers to yeasts might promote K^+^ efflux, extracellular acidification, inhibition of Rb^+^ uptake, increased transmembrane potential difference, and increased uptake of Ca^2+^, thus causing the inhibition of some metabolic pathways such as respiration and fermentation [61].

## 5 Conclusion

In our previous studies [29], chitosan-shelled/decafluoropentane-cored oxygenloaded nanodroplets (OLNs) have proven effective in delivering oxygen to hypoxic tissues and ultrasound-triggered transdermal delivery-activated chitosan-shelled OLNs appear as promising, nonconventional and innovative tools for adjuvant treatment of infected chronic wounds

Furthermore, ultrasound-activated chitosan-shelled OLNs appeared as promising, nonconventional and innovative tools for adjuvant treatment of infected chronic wounds.

All the results provided in the present study support the hypothesis that chitosan-shelled and perfluoropentane-filled OLNBs appear as innovative and promising tools to treat CWs, since they are non-toxic and cost-effective devices which not only promote repair processes in human skill cells after prolonged exposure to hypoxic conditions, but also display anti-microbial properties against microorganisms such as MRSA and *C. albicans* that are frequently associated with CWs. Therefore, the present results suggest that OLNBs should deserve undergoing deeper investigation to verify their potential employment in future treatment of infected CWs in the elderly.

## Conflict of interest

The authors declare no conflict of interest.

## Declaration of Competing Interest

The authors report no declarations of interest.
